# Stability of pro- and anti-inflammatory immune biomarkers for human cohort studies

**DOI:** 10.1186/s12967-017-1154-3

**Published:** 2017-03-02

**Authors:** C. Graham, R. Chooniedass, W. P. Stefura, L. Lotoski, P. Lopez, A. D. Befus, A. B. Becker, K. T. HayGlass

**Affiliations:** 1 Department of Immunology, University of Manitoba, Winnipeg, MB Canada; 2 Department of Pediatrics and Child Health, University of Manitoba, Winnipeg, MB Canada; 3 Children’s Hospital Research Institute of Manitoba, Winnipeg, MB Canada; 4 Division of Pulmonary Medicine, Department of Medicine, University of Alberta, Edmonton, AB Canada; 5 Community Health and Epidemiology-‎Saskatchewan Population Health and Evaluation Research Unit, University of Saskatchewan, Saskatoon, SK Canada

**Keywords:** Cohort, Inflammation, Human immunology, Immune regulation, Innate, Allergy, Autoimmunity

## Abstract

**Background:**

Although discovery research has identified the importance of dozens of pro- and anti-inflammatory immune mediators in the pathogenesis, maintenance, exacerbation and resolution of inflammatory diseases, most human cohort studies have incorporated few or no immunological intermediate phenotypes in their analyses. Significant hindrances have been (1) the limited panel of biomarkers known to be readily detected in healthy human populations and (2) the stability, hence utility, of such biomarkers to repeated analysis.

**Methods:**

The frequency and stability of 14 plasma biomarkers linked to in vivo immune regulation of allergic and autoimmune inflammatory disorders was determined in 140 healthy pediatric and adult participants. The impact of initial and multiple subsequent freeze/thaw cycles on pro-inflammatory (CCL2, CXCL10, IL-18, TNFα, IL-6), anti-inflammatory (IL-10, sTNF-RII, IL-1Ra), acute phase proteins (CRP, PTX3) and other biomarkers (sST2, IL-1RAcP) was subsequently quantified.

**Results:**

Multiple biomarkers capable of providing an innate immune signature of inflammation were readily detected directly ex vivo in healthy individuals. These biomarker levels were unaffected when comparing paired data sets from freshly obtained, never frozen plasma or serum and matched aliquots despite extensive freeze/thaw cycles. Neither age nor sex affected stability. Similarly, no quantitative differences were found following repetitive analysis of inflammatory biomarkers in culture samples obtained following in vitro stimulation with TLR and RLR ligands.

**Conclusions:**

A broad panel of in vivo and ex vivo cytokine, chemokine and acute phase protein biomarkers that have been linked to human chronic inflammatory disorders are readily detected in vivo and remain stable for analysis despite multiple freeze thaw cycles. These data provide the foundation and confidence for large scale analyses of panels of inflammatory biomarkers to provide better understanding of immunological mechanisms underlying health versus disease.

**Electronic supplementary material:**

The online version of this article (doi:10.1186/s12967-017-1154-3) contains supplementary material, which is available to authorized users.

## Background

Cohorts that range from hundreds to tens of thousands of individuals offer a powerful tool to validate, integrate and extend multidisciplinary findings obtained in basic biomedical discovery research. Currently, many human cohorts focus on identifying correlates of environmental variables (i.e. endotoxin exposure, birth order) with clinical outcomes. With few exceptions [1–3], only a small proportion of such publications provide detailed examination of the intermediate phenotype of in vivo cytokine responses. For example, for allergic diseases, the most common chronic immune disorder in humans, PubMed identifies over 2000 publications for “birth cohort and allergy,” but <10% (155 citations) incorporate “cytokine or IL*,” and of these, only a subset examine inflammatory cytokines. Recent meta-analyses identify the knowledge gap resulting from this omission [4–8]. Given that most allergic and autoimmune disorders are driven by dysfunctional immune regulation, this identifies an important, but often missed, opportunity to enhance understanding of endotypes and mechanisms [9–12] underlying inflammatory diseases. The importance of doing so is increasingly clear [13–22].

A major logistical challenge that impedes more widespread adoption of such analyses is continuing uncertainty about the stability of immune biomarkers in plasma, serum or tissue culture samples to repeated analysis. Repetitive freezing and thawing (F/T) cycles can induce protein instability and aggregation [23, 24]. Among immune biomarkers in complex biological fluids (i.e. plasma, culture supernatants), some studies indicate extreme sensitivity, while others report that minimal variance results from the limited number of F/T cycles that are typically required. This controversy may arise from the limited number of cytokine biomarkers that have been examined to date, the small sample sizes often utilized (i.e. frequently < 10 individuals), the fact that several biomarkers examined are typically undetectable or at the limits of assay sensitivity in much of the healthy population examined (i.e. IL-6), hence substitution of spiked recombinant commercial proteins occurs as a surrogate for in vivo biomarkers. To an even greater extent than discovery based research projects which examine 20–40 individuals, cohorts face extensive logistical difficulties that prohibit analyses without repeated cycles of F/T [25–27].

Here we test the hypothesis that exposure of immune pro- and anti-inflammatory biomarkers to at least five repeated F/T cycles introduces acceptable (i.e. minimal and predictable) variation. Using a panel of 14 pro-inflammatory (CCL2, CXCL10, IL-18, TNFα, IL-6), anti-inflammatory (IL-10, sTNF-RII, IL-1Ra), acute phase proteins (CRP, PTX3) and other biomarkers (sST2, IL-1RAcP) of inflammatory disorders previously linked to asthma, other atopic disorders [28–31] and a wide range of other chronic inflammatory conditions in humans, we quantify the sensitivity to initial freezing as well as to repeated F/T cycles that are inevitable if large studies incorporate analyses of immunological intermediate phenotypes. The findings demonstrate that a broad panel of pro-inflammatory, anti-inflammatory, acute phase proteins and other biomarkers of inflammatory diseases are readily amenable to analysis and should be more widely incorporated in large human cohort studies.

## Methods

### Participants

The Canadian Healthy Infant Longitudinal Development (CHILD) Study is a prospective longitudinal birth cohort of >3500 neonates [32]. In this report, following approval by the University of Manitoba Health Research Ethics Board, and written informed consent from each participant or their parent/guardian, non-fasting venous blood was obtained at the Winnipeg site to yield plasma and serum samples from 140 randomly selected participants (children and their parents).

### Sample preparation

Peripheral blood was collected by venipuncture and used for plasma, serum and isolation of PBMC [33]. Briefly, samples were kept at room temperature during transport and prior to processing. Plasma was collected from 10-mL heparin Vacutainer tubes (BD, Mississauga, Canada) by centrifugation (500*g*, 10 min). Serum was collected from 6-mL silica-coated Vacutainer tubes (BD) by centrifugation (1000*g*, 10 min). Replicate plasma and serum sample aliquots (300 μL each) were prepared from each individual and then used within 24 h without freezing and were frozen at −80 °C for one or five F/T cycles before analysis. All analyses were performed comparing paired samples from the same individuals. Because the goal was to determine the impact of repeated changes of state (i.e. freeze/thawing) on plasma and supernatant samples, rather than how many months or years a sample could be stored and retain its integrity, all samples that underwent freeze thaw cycles were carried out within 24 h when comparing never frozen plasma or culture supernatant with samples subjected to one or five F/T cycles. Paired analyses of one versus five F/T cycles were carried out with storage at −80 °C for a few days to a month in total. Samples were handled using standard laboratory conditions: thawed rapidly at 37 °C then kept on ice until analyzed.

### PBMC isolation and cell culture

PBMC were prepared using Ficoll (GE Healthcare, Mississauga, Canada) and cultured (triplicates, 350,000 cells/round bottom well in 200 μL, 24 h) in medium alone or with stimuli. Medium consisted of RPMI-1640 (Thermo Fisher Scientific, Mississauga, Canada) supplemented with 10% fetal bovine serum (GE Healthcare, Mississauga, Canada), 1% l-glutamine (VWR International, Mississauga, Canada), 1% Antibiotic–Antimycotic (Thermo Fisher Scientific), and 0.1% 2-mercaptoethanol (Thermo Fisher Scientific). Innate stimuli used included TLR4 ligand LPS (0.4 ng/mL, InvivoGen, San Diego, CA) or RLR ligand Poly(I:C)/Lyovec (250 ng/mL, InvivoGen). All PBMC samples were cultured the day they were drawn. Supernatants were aliquoted and examined in parallel without ever freezing and after five F/T cycles at −80 and 37 °C.

### Immunological assays

All analyses were carried out in duplicate with paired samples after 0–5 F/T cycles. 5% of sample pairs or triplets were repeated on a separate day. Meso Scale Discovery (MSD, Rockville, Maryland) singleplex assays were used to analyze plasma, serum and culture supernatants for CCL2, CRP, CXCL8, IL-6, IL-10, IL-18, IL-1Ra, sTNF-RII and TNFα according to manufacturer`s instructions. MSD V-Plex assays were used to analyze plasma and supernatant levels of CXCL10. ELISA (alkaline phosphatase-biotin coupled developing reagent with PNPP for development) was used to analyze plasma and serum levels of IL-1RAcP, PTX3, and sST2 (R&D Systems, Minneapolis, Minnesota) using ultrasensitive protocols as previously described [34]. ELISAs incorporated four serial dilutions of each sample (i.e. 1/2, 1/4, 1/8 and 1/16) that were assessed against eight serial dilutions of fresh aliquots of a constant recombinant lab standard stored at −80 °C in individual 400 μL aliquots (Cedarlane, Burlington, Canada; PeproTech, Quebec, Canada; R&D Systems). In most experiments, median coefficients of intra-assay variation between assays were below 5% for MSD assays and 10–15% for ELISA. Inter-assay variation was typically <10–20%.

### Statistics

Data were analyzed using GraphPad Prism (La Jolla, California). Each point represents a single sample from an individual aliquot that has undergone the indicated number of F/T cycles. Mann–Whitney or Wilcoxon Matched Pairs/Signed Rank tests were used for unpaired and paired data sets respectively. While the multiple comparisons used in this study would normally require use of Bonferroni corrections, to obtain maximum sensitivity for detection of possible differences, significance was assessed at the lower threshold of a 95% confidence level (two-tailed p < 0.05).

## Results

### Detection and stability of pro- and anti-inflammatory plasma biomarkers

Freshly obtained plasma (or sera, see below) from a study population of 140 individuals of whom 30 were pediatric were examined. Adults were 20–44 years of age (median 32 year), while the pediatric population was aged 3 (median 3.2 year). Due to the number of biomarkers examined, and logistical constraints on how many independent assays could be performed simultaneously with never frozen samples, slightly different numbers of individuals were examined for each biomarker.

**Fig. 1:**
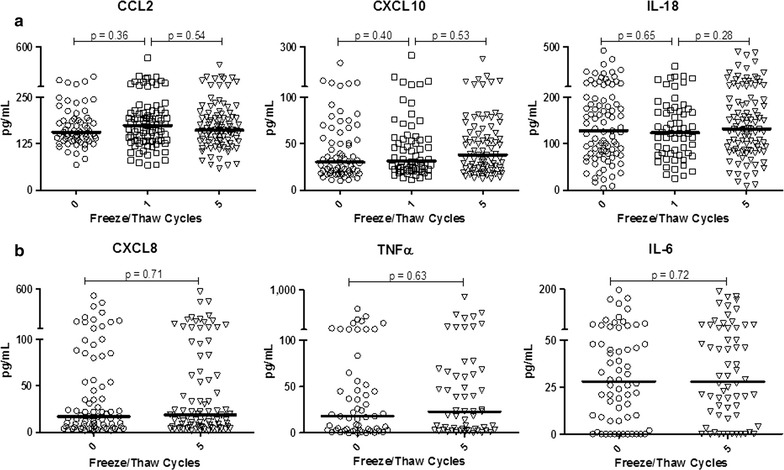
Initial freezing, and up to five total freeze/thaw cycles, does not alter the levels of pro-inflammatory cytokines and chemokines in plasma. *Each symbol* represents an independent sample from a separate individual. Medians and p values from Wilcoxon matched pairs analyses are shown

Figure 1a demonstrates that pro-inflammatory cytokines CCL2, CXCL10 and IL-18 are expressed at readily detectable levels in vivo in all of the individuals examined and can serve as readily detectable plasma biomarkers in healthy human populations. When comparing fresh plasma with that from the same individuals after zero, one, or five F/T cycles (see “Methods” section), there was no detectable impact on the concentrations measured. Similarly, CXCL8, TNFα and IL-6 values in never frozen plasma versus 5 × F/T samples were indistinguishable (p > 0.05) in paired longitudinal analyses (Fig. 1b).

**Fig. 2:**
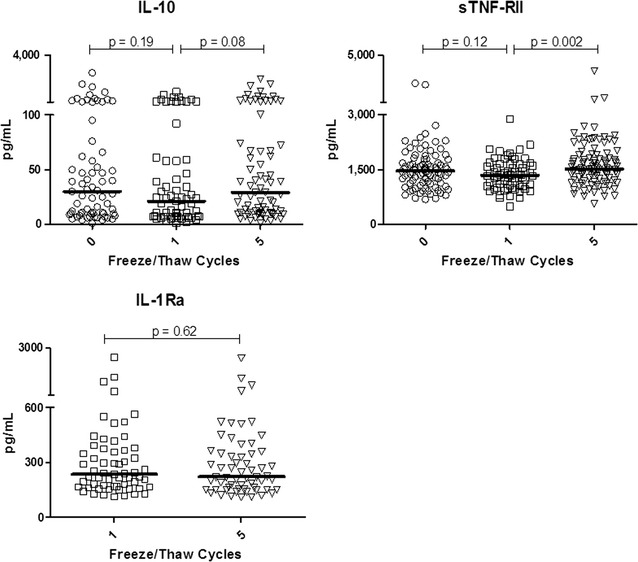
Plasma anti-inflammatory biomarkers are stable despite repeated freeze/thaw cycles

Endogenous controls of inflammation, such as anti-inflammatory cytokines and immune response modifiers, are pivotal yet are typically underrepresented in human studies of inflammation. Figure 2 reveals three points. Unlike pro-inflammatory biomarkers such as IL-6 and TNFα, where many healthy individuals exhibit extremely low levels, IL-10, sTNF-RII and IL-1Ra are readily detected in plasma of most individuals, hence can be readily measured in cohort studies. Secondly, an initial F/T cycle, virtually inevitable in practice, does not alter the results obtained upon analysis. These data are also presented in before/after plots (Additional file 1: Figure S1) to facilitate intra-individual comparisons. Finally, particularly important for studies where hundreds or thousands of samples require assessment, up to five F/T cycles had minimal impact on the levels measured.

A single exception was found among the 14 biomarkers examined. sTNF-RII differed when comparing 1× versus 5× F/T cycles (medians 1516 vs. 1470 pg/mL, a 3% increase, p = 0.002). We note that comparison of never frozen plasma and 5× F/T was *not* significant (medians 1470 vs. 1516 pg/mL, p = 0.14). For context, the 3% difference seen comparing one and five F/T cycles contrasts with the 770% range (560–4365 pg/mL) in the population. Thus, while the 1× versus 5× F/T is statistically significant (if it had not been corrected for multiple comparisons, as would normally be performed), the biological relevance of a median 3% error in analysis elicited by freezing and thawing is minor in comparison to a 7.7 fold range in the population.

### Stability of systemic inflammatory biomarkers: CRP, PTX3, IL-1RAcP and sST2

**Fig. 3:**
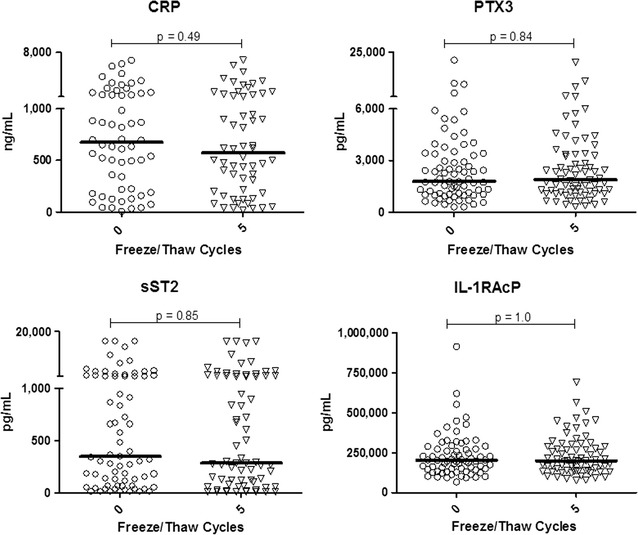
Stability of acute phase proteins and related plasma inflammatory biomarkers to at least five freeze/thaw cycles

We next examined a panel of widely used and emerging biomarkers of acute and chronic inflammation in humans (Fig. 3). These included CRP, historically the most commonly examined acute phase protein biomarker in humans; pentraxin 3 (PTX3), a systemic acute phase protein expressed in vivo more rapidly than CRP and prominent in chronic inflammation; soluble IL-1R Accessory protein (IL-1RAcP), a widely expressed protein integral to pro-inflammatory signaling and linked to asthma, autoimmunity and other inflammatory conditions; and sST2, an increasingly used predictive biomarker of inflammatory disorders and increased risk of mortality in cardiovascular disease. All were found to be ubiquitously expressed in plasma of healthy individuals. Each of these biomarkers was also stable when comparing paired data sets from freshly obtained, never frozen plasma and matched aliquots after five F/T cycles.

### Serum versus plasma stability to repeated F/T handling

**Fig. 4:**
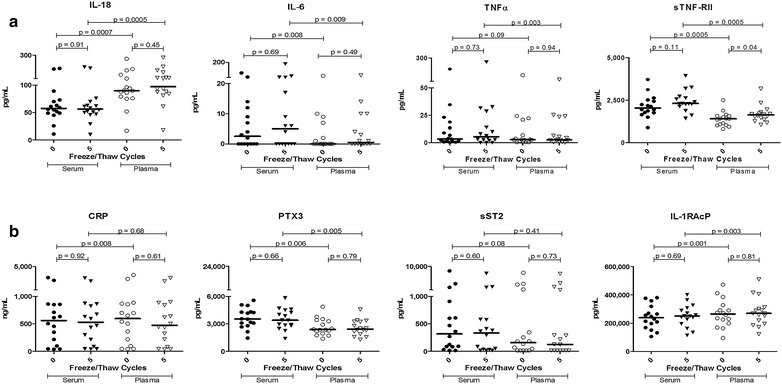
Pro- and anti-inflammatory cytokines (**a**), acute phase proteins and inflammatory biomarkers (**b**) in serum, as well as plasma, remain stable despite repeated freeze/thaw cycles but the values obtained from the two sources are often distinct. Serum and plasma were obtained from a set of individuals and analysed both immediately and following five freeze/thaw cycles

The utility of plasma versus serum in biomarker quantification has been extensively studied. Here we focus on F/T stability of serum, examining a much broader range of immune analytes than previously reported. When serum was obtained from an individual and subjected to zero versus five repeat F/T cycles, the values obtained were stable. This conclusion applied to pro- and anti-inflammatory cytokines (Fig. 4a) as well as acute phase proteins and other biomarkers (Fig. 4b). Importantly, and as previously established, levels for a given individual in serum versus plasma are frequently independent and are not necessarily interchangeable. Thus, paired analyses of serum and plasma from the same donors frequently yielded different values for some biomarkers whereas levels of other biomarkers were indistinguishable in serum and plasma.

### Impact of donor age or sex on F/T stability

**Fig. 5:**
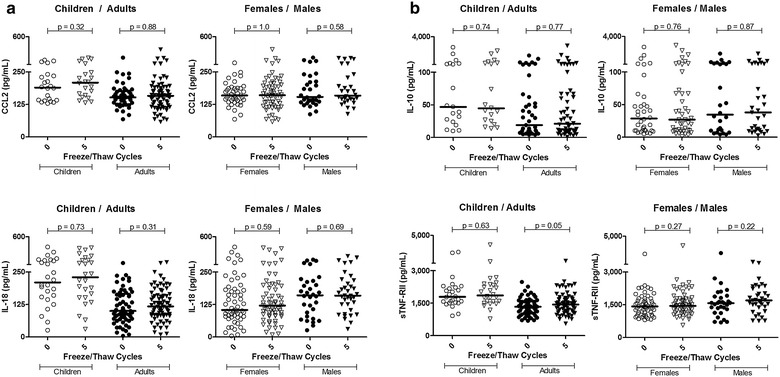
The stability of pro-inflammatory (**a**) and anti-inflammatory (**b**) plasma biomarkers is similar in both pediatric and adult populations. Sex does not alter the freeze/thaw stability of these plasma biomarkers

Conclusions from experimental or clinical findings are sometimes extended from one age group or sex to another in the absence of clear supporting evidence. To avoid this, we stratified the study population by age, comparing samples within young children independently from those of adults (median age 3 vs. 32). Figure 5 demonstrates that F/T stability for the two pro-inflammatory (CCL2, IL-18; Fig. 5a) and two anti-inflammatory (IL-10, sTNF-RII; Fig. 5b) molecules shown applies equally to pediatric populations. Similarly, male versus female donors did not exhibit sex-attributable differences in stability of these biomarkers. CRP, PTX3, IL-1RAcP (encoded by *IL1RAP*) and sST2 (encoded by *IL1RL1*) were also stable within age and sex stratifications when comparing never frozen samples with those of five F/T cycles.

### Stability of PRR-stimulated responses generated in primary culture

**Fig. 6:**
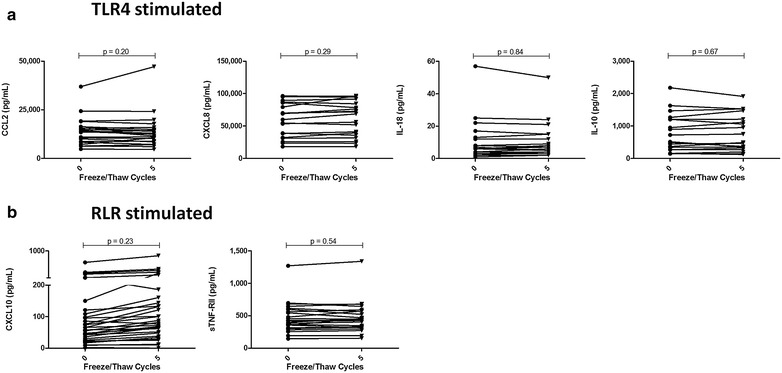
Stability of pro- and anti-inflammatory biomarkers following acute PRR-mediated [TLR4 (**a**), RLR (**b**)] stimulation in vitro

Acute PRR-stimulated innate immune responses provide additional insight into immune capacity. Here, primary culture supernatants were generated by stimulation of fresh PBMC with agonists for a representative TLR (TLR4 using LPS, n = 20 experiments, Fig. 6a) or RLR (RIG-I/MDA-5 using Lyovec conjugated poly:IC, n = 30 experiments, Fig. 6b) ligand. Both pro- and anti-inflammatory responses were stable for at least five F/T cycles.

## Discussion

Human inflammatory disease research is hampered by use of a relatively small number of biomarkers to translate findings from basic biomedical research into large scale cohorts. Here we demonstrate a panel of 14 in vivo biomarkers of pro- and anti-inflammatory status that are readily quantifiable in plasma of healthy adult and pediatric populations. The results also demonstrate that no differences are evident when comparing fresh/never frozen samples with those that had undergone up to five F/T cycles. Similarly, a panel of biomarkers of innate PRR-mediated activation remained stable in supernatants obtained after cell culture stimulation, directly ex vivo, despite repeated freeze thaw cycles. Thus, extending findings from individual murine and human analyses of inflammatory disorders to large human cohorts by obtaining more comprehensive innate immune signatures is readily feasible.

Prior literature on F/T stability has yielded contradictory conclusions. A representative early study (with four healthy and three HIV-infected volunteers) indicated F/T stability for the biomarkers examined [35] as did similar studies [36, 37]. Others disagreed, finding substantial sensitivity to F/T [38–41]. Using four healthy individuals, De Jager et al. [27] concluded that samples for cytokine measurements could not be subjected to repeated F/T cycles because only two of the 15 cytokines they examined did not show alterations in mean levels.

Important caveats to be aware of in interpretation of the literature include: (1) due to limited assay sensitivity, many investigators utilized samples spiked with recombinant cytokines to achieve sufficient sensitivity for the assays employed, (2) the number of individuals studied in most studies was often less than ten, (3) intra- and inter-assay variation was often not provided, making it difficult to determine to what extent decreases or increases in reported cytokine levels were attributable to variability in assay or operator performance rather than F/T cycles. Moreover, in many studies, means and parametric statistical tests with significance set at 0.05 were utilized, without correction for non-parametric data distributions (log-transformation or use of non-parametric tests such as utilized above) common to small data sets or correcting for multiple comparisons (i.e. Bonferroni corrections).

When statistically significant differences are identified, it will be important to examine the scale of such differences in the context of the population being examined. For example, here, sTNF-RII levels were significantly different following one versus five F/T cycles (medians 1516 vs. 1470 pg/mL, a 3% increase, p = 0.002). While variance is inherent in repeated analyses of any quantitative measure, it needs to be compared in scale to the range exhibited within the study population as a whole. Thus, for sTNFRII, there is a 770% (560–4365 pg/mL) range in values within the population studied. Similarly, the ranges of CXCL10 (2500% or 25-fold range between weakest and strongest), IL-18 (>125 fold), TNFα (>1000 fold) and IL-10 (>3000 fold) seen in this relatively small human population (n = 140) are important to weigh in assessing biological as well as statistical significance even when a small, statistically significant difference is contributed by sample handling.

This study has important caveats. The focus here was on the capacity to reproducibly quantify pro-/anti-inflammatory biomarker concentrations ex vivo, despite virtually inevitable F/T cycles. We did not attempt to determine biological activity (i.e. therapeutic potential), nor did we assess the stability of recombinant proteins in these assays. Other (largely controllable) factors can introduce sample variability and, depending on the study design utilized, need to be considered individually. Similarly, immune biomarkers such as Type I or III Interferons (18) that were not examined in this study may exhibit sensitivity to F/T cycles. As additional biomarkers are added, it will be important to examine each explicitly prior to undertaking large-scale analyses. Other controllable variables, including operator error, assay variance and so forth may also introduce variance. This underlines the need for well-defined standard operating procedures (SOPs). Finally, proving that something does not occur is impossible. For that reason, this study utilized well over 100 different individuals. Variation might become detectable if 1000 or 100,000 individuals were examined, but if thousands or more samples are required to detect a difference, the size of that effect would by definition be minor.

One concern not addressed here is the long term stability of biomarkers after years of storage. Addressing this variable was beyond the scope of the present study. Use of cross-sectional study designs when comparing samples in a longitudinal cohort after three, five or seven years of storage provide an interim workaround until better data on long term stability are available. Thus, comparison can be made of samples of the same age from individuals exhibiting versus not exhibiting a given clinical phenotype is feasible, if imperfect. Certainly, prioritizing analyses to the earliest possible time point is important.

Finally, while commonly known, an important practical aspect in the implementation of such analyses should be reiterated. Logistical constraints on assay manufacturers preclude provision of constant standards from one assay to another purchased a few months or years later. This underlines the importance of establishing substantial aliquots of a single internal lab standard to be used for each assay to allow inter-assay comparison.

## Conclusions

Human cohorts generate tens of thousands of biological samples, often at multiple sites. It is impossible to examine samples for all analytes simultaneously. The results above provide the foundation (and confidence) for large scale analyses of panels of inflammatory biomarkers to better understand immunological mechanisms underlying health versus disease. Specifically, the data demonstrate that an extensive panel of pro-inflammatory (CCL2, CXCL10, IL-18, TNFα, IL-6), anti-inflammatory (IL-10, sTNF-RII, IL-1Ra), acute phase proteins (CRP, PTX3) and other biomarkers (sST2, IL-1RAcP) linked to allergy and autoimmunity and other inflammatory diseases in basic discovery research are readily detectable, even in healthy control individuals, and that they remain stable for repeat analysis despite multiple freeze thaw cycles. More frequent and more comprehensive examination of innate immune signatures would greatly enhance the value obtained from large multi-centre human cohort studies.

## Additional file

**Additional file 1: Figure S1.** Data from representative biomarkers shown Figs. 1, 2 and 3 portrayed in a before/after format.

## Abbreviations

F/T: freeze/thaw
PBMC: peripheral blood mononuclear cells
PRR: pattern recognition receptor
RLR: RIG-like receptors
TLR: toll like receptor
