# Supplementary figures and images for: Stability of pro- and anti-inflammatory immune biomarkers for human cohort studies
Source: J Transl Med. 2017 Mar 2;15:53. doi: 10.1186/s12967-017-1154-3 (PMC5335797; doi:10.1186/s12967-017-1154-3)

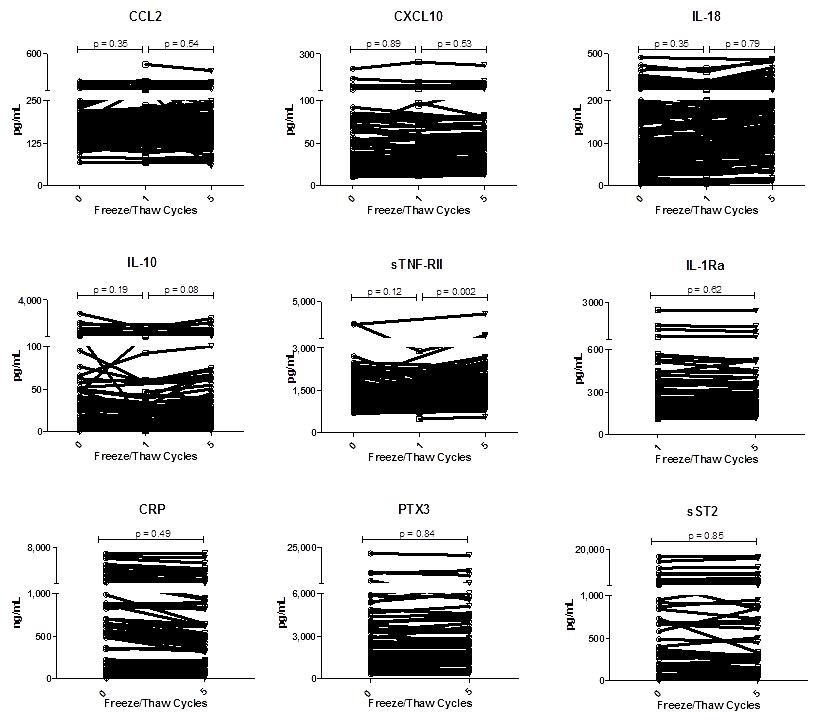

Supplement: Supplementary file 1 — Additional file 1: Figure S1. Data from representative biomarkers shown Figs. 1, 2 and 3 portrayed in a before/after format. [file 12967_2017_1154_MOESM1_ESM.tif]
